# Supplementary material for: Rapid transgenerational adaptation in response to intercropping reduces competition
Source: eLife. 2022 Sep 13;11:e77577. doi: 10.7554/eLife.77577 (PMC9553216; doi:10.7554/eLife.77577)
Supplement: Supplementary file 1. — (a) Type-I Analysis of Variance Table of the experimental treatment effects on net, competition and facilitation indexes (RII), in year 3 (2020). (b) Pairwise comparisons of the effect on net interaction index (RII) between fertilizer (yes, no), coexistence history (diff [different], same), and monoculture vs mixture (mix [mixture], mono [monoculture]). (c) Type-I Analysis of Variance Table of the experimental treatment effects on net, complementarity, and selection effects in year 3 (2020). (d) Pairwise comparisons of the effect on net biodiversity effects between fertilizer (yes, no) and coexistence history (diff [different], same). (e) Pairwise comparisons of the effect on selection effects between fertilizer (yes, no), coexistence history (diff [different], same), and planted diversity (2 vs 4). (f) Type-I Analysis of Variance Table of the experimental treatment effects on total crop yield per plot (square-root transformed). (g) Type-I Analysis of Variance Table of the experimental treatment effects on mean and coefficient of variation of height, per species per plot (species level) in year 3 (2020). (h) Type-I Analysis of Variance Table of the experimental treatment effects on mean and coefficient of variation of width, per species per plot (species level) in year 3 (2020). (i) Type-I Analysis of Variance Table of the experimental treatment effects on mean and coefficient of variation of SLA, per species per plot (species level) in year 3 (2020). (j) Type-I Analysis of Variance Table of the experimental treatment effects on mean and coefficient of variation of LDMC, per species per plot (species level) in year 3 (2020). (k) Type-I Analysis of Variance Table of the experimental treatment effects on mean and coefficient of variation of mass per seed, per species per plot (species level) in year 3 (2020). (l) Type-I Analysis of Variance Table of the experimental treatment effects on community-weighted mean and coefficient of variation of height, per plot (communit [file elife-77577-supp1.docx]

**Supplementary File 1**

**Supplementary file 1a**. **Type-I Analysis of Variance Table of the experimental treatment effects on net, competition and facilitation indexes (RII), in year 3 (2020)**

*DenDF*, degrees of freedom of error term; *NumDF*, degrees of freedom of term; *F-value*, variance ratio; *Pr(>F)*, error probability. P-values in bold are signiﬁcant at α = 0.05; * (P < 0.05), ** (P < 0.01), *** (P < 0.001). n =276

|  |  | *Net RII* | | |
| --- | --- | --- | --- | --- |
|  | *NumDF* | *DenDF* | *F value* | *Pr(>F)* |
| *Fertilizer* | 1 | 6.985 | 44.4907 | **0.000288 ***** |
| *History* | 1 | 229.382 | 30.4138 | **9.38E-08 ***** |
| *Monocultures vs. mixtures* | 1 | 19.988 | < 1 | 0.458208 |
| *Diversity* | 1 | 19.987 | < 1 | > 0.5 |
| *Fertilizer x history* | 1 | 229.409 | < 1 | > 0.5 |
| *Fertilizer x mono vs. mix* | 1 | 243.671 | < 1 | > 0.5 |
| *Fertilizer x diversity* | 1 | 242.181 | < 1 | > 0.5 |
| *History x mono vs. mix* | 1 | 241.923 | 3.2799 | 0.071375 |
| *History x diversity* | 1 | 242.397 | < 1 | 0.323169 |
| *Fertilizer x history x mono vs. mix* | 1 | 241.909 | < 1 | > 0.5 |
| *Fertilizer x history x diversity* | 1 | 242.383 | < 1 | > 0.5 |

**Supplementary file 1b**. **Pairwise comparisons of the effect on net interaction index (RII) between fertilizer (yes, no), coexistence history (diff [different], same), and monoculture vs mixture (mix [mixture], mono [monoculture]).**

| Net interaction index | estimate | SE | df | t.ratio | p.value |
| --- | --- | --- | --- | --- | --- |
| no diff mix - yes diff mix | 0.220655 | 0.0552 | 42.5 | 4 | 0.0056 |
| **no diff mix - no same mix** | -0.28115 | 0.068 | 228.3 | -4.133 | 0.0013 |
| no diff mix - yes same mix | -0.00095 | 0.0674 | 77.7 | -0.014 | > 0.5 |
| no diff mix - no diff mono | 0.128584 | 0.2139 | 22.9 | 0.601 | > 0.5 |
| no diff mix - yes diff mono | 0.325046 | 0.214 | 22.9 | 1.519 | > 0.5 |
| no diff mix - no same mono | 0.028404 | 0.221 | 26 | 0.129 | > 0.5 |
| no diff mix - yes same mono | 0.255199 | 0.2208 | 26 | 1.156 | > 0.5 |
| yes diff mix - no same mix | -0.5018 | 0.0678 | 70.8 | -7.405 | <.0001 |
| **yes diff mix - yes same mix** | -0.2216 | 0.0671 | 243.8 | -3.302 | 0.0241 |
| yes diff mix - no diff mono | -0.09207 | 0.214 | 22.9 | -0.43 | > 0.5 |
| yes diff mix - yes diff mono | 0.104391 | 0.2138 | 22.9 | 0.488 | > 0.5 |
| yes diff mix - no same mono | -0.19225 | 0.2209 | 26 | -0.87 | > 0.5 |
| yes diff mix - yes same mono | 0.034544 | 0.2207 | 26 | 0.157 | > 0.5 |
| no same mix - yes same mix | 0.280204 | 0.0781 | 102.2 | 3.588 | 0.0116 |
| no same mix - no diff mono | 0.409733 | 0.2176 | 24.4 | 1.883 | > 0.5 |
| no same mix - yes diff mono | 0.606195 | 0.2175 | 24.4 | 2.786 | 0.1447 |
| no same mix - no same mono | 0.309553 | 0.224 | 27.6 | 1.382 | > 0.5 |
| no same mix - yes same mono | 0.536347 | 0.2243 | 27.6 | 2.391 | 0.2846 |
| yes same mix - no diff mono | 0.129529 | 0.2175 | 24.4 | 0.596 | > 0.5 |
| yes same mix - yes diff mono | 0.325991 | 0.2174 | 24.4 | 1.5 | > 0.5 |
| yes same mix - no same mono | 0.029349 | 0.2243 | 27.6 | 0.131 | > 0.5 |
| yes same mix - yes same mono | 0.256143 | 0.224 | 27.6 | 1.143 | > 0.5 |
| no diff mono - yes diff mono | 0.196462 | 0.0786 | 109.6 | 2.498 | 0.2074 |
| **no diff mono - no same mono** | -0.10018 | 0.0959 | 244 | -1.044 | > 0.5 |
| no diff mono - yes same mono | 0.126614 | 0.0958 | 169.5 | 1.321 | > 0.5 |
| yes diff mono - no same mono | -0.29664 | 0.0958 | 170.5 | -3.096 | 0.0463 |
| **yes diff mono - yes same mono** | -0.06985 | 0.0953 | 239.9 | -0.733 | > 0.5 |
| no same mono - yes same mono | 0.226794 | 0.1104 | 205.3 | 2.055 | 0.4476 |

**Supplementary file 1c**. **Type-I Analysis of Variance Table of the experimental treatment effects on net, complementarity, and selection effects in year 3 (2020)**

*DenDF*, degrees of freedom of error term; *NumDF*, degrees of freedom of term; *F-value*, variance ratio; *Pr(>F)*, error probability. P-values in bold are signiﬁcant at α = 0.1; . (P < 0.1); * (P < 0.05), ** (P < 0.01), *** (P < 0.001). n=204

|  |  | *Net effect* | | | *Complementarity effect* | | | *Selection effect* | | |
| --- | --- | --- | --- | --- | --- | --- | --- | --- | --- | --- |
|  | *NumDF* | *DenDF* | *F value* | *Pr(>F)* | *DenDF* | *F value* | *Pr(>F)* | *DenDF* | *F value* | *Pr(>F)* |
| *Fertilizer* | 1 | 7.64 | 1.005 | 0.3468 | 7.38 | 2.684 | 0.14312 | 7.69 | 1.295 | 0.2894 |
| *History* | 1 | 177.34 | < 1 | > 0.5 | 179.89 | 1.567 | 0.2123 | 178.62 | 1.512 | 0.2204 |
| *Diversity* | 1 | 14.90 | 2.583 | 0.1290 | 14.87 | 5.887 | **0.0285 *** | 15.06 | 4.009 | **0.0636 .** |
| *Fertilizer x history* | 1 | 177.08 | 9.595 | **0.0023 **** | 179.80 | 2.719 | 0.1009 | 178.66 | 2.498 | 0.1158 |
| *Fertilizer x diversity* | 1 | 173.48 | < 1 | > 0.5 | 174.92 | 5.579 | **0.0193 *** | 178.22 | 6.092 | **0.0145 *** |
| *History x diversity* | 1 | 173.66 | < 1 | > 0.5 | 175.06 | 1.399 | 0.2385 | 178.42 | 3.165 | **0.0769 .** |
| *Fertilizer x history x diversity* | 1 | 173.36 | < 1 | > 0.5 | 174.85 | 2.026 | 0.1564 | 178.35 | 4.093 | **0.0446 *** |

**Supplementary file 1d**. **Pairwise comparisons of the effect on net biodiversity effects between fertilizer (yes, no) and coexistence history (diff [different], same).**

| Net biodiversity effects | estimate | SE | df | t.ratio | p.value |
| --- | --- | --- | --- | --- | --- |
| no diff - yes diff | 2.1 | 8.29 | 11.6 | 0.254 | > 0.5 |
| **no diff - no same** | 11.73 | 6.19 | 176.4 | 1.893 | 0.2347 |
| no diff - yes same | -13.19 | 8.94 | 15.5 | -1.476 | 0.4742 |
| yes diff - no same | 9.62 | 9.05 | 16.1 | 1.063 | > 0.5 |
| **yes diff - yes same** | -15.3 | 6.04 | 173.4 | -2.531 | 0.0587 |
| no same - yes same | -24.92 | 9.65 | 20.5 | -2.583 | 0.0763 |

**Supplementary file 1e**. **Pairwise comparisons of the effect on selection effects between fertilizer (yes, no), coexistence history (diff [different], same), and planted diversity (2 vs 4).**

| Selection effects | estimate | SE | df | t.ratio | p.value |
| --- | --- | --- | --- | --- | --- |
| no diff diversity2 - yes diff diversity2 | 1.061 | 5.09 | 18.7 | 0.209 | > 0.5 |
| no diff diversity2 - no same diversity2 | 0.754 | 5.74 | 179.3 | 0.131 | > 0.5 |
| no diff diversity2 - yes same diversity2 | -1.453 | 6.07 | 33.3 | -0.239 | > 0.5 |
| no diff diversity2 - no diff diversity4 | 1.35 | 8 | 50.8 | 0.169 | > 0.5 |
| no diff diversity2 - yes diff diversity4 | 32.189 | 8.26 | 41.7 | 3.899 | 0.0076 |
| no diff diversity2 - no same diversity4 | 4.08 | 10.01 | 99.2 | 0.408 | > 0.5 |
| no diff diversity2 - yes same diversity4 | -1.131 | 10.1 | 82.2 | -0.112 | > 0.5 |
| yes diff diversity2 - no same diversity2 | -0.307 | 6.03 | 34.8 | -0.051 | > 0.5 |
| yes diff diversity2 - yes same diversity2 | -2.514 | 5.73 | 179.5 | -0.439 | > 0.5 |
| yes diff diversity2 - no diff diversity4 | 0.289 | 8.24 | 41.4 | 0.035 | > 0.5 |
| yes diff diversity2 - yes diff diversity4 | 31.128 | 7.98 | 49 | 3.902 | 0.0066 |
| yes diff diversity2 - no same diversity4 | 3.02 | 10.16 | 80.1 | 0.297 | > 0.5 |
| yes diff diversity2 - yes same diversity4 | -2.192 | 9.86 | 97.2 | -0.222 | > 0.5 |
| no same diversity2 - yes same diversity2 | -2.207 | 6.92 | 50.6 | -0.319 | > 0.5 |
| no same diversity2 - no diff diversity4 | 0.596 | 8.68 | 65.9 | 0.069 | > 0.5 |
| no same diversity2 - yes diff diversity4 | 31.434 | 8.86 | 54.1 | 3.547 | 0.0172 |
| no same diversity2 - no same diversity4 | 3.326 | 10.35 | 109.2 | 0.321 | > 0.5 |
| no same diversity2 - yes same diversity4 | -1.885 | 10.61 | 94.4 | -0.178 | > 0.5 |
| yes same diversity2 - no diff diversity4 | 2.803 | 8.86 | 52.9 | 0.316 | > 0.5 |
| yes same diversity2 - yes diff diversity4 | 33.641 | 8.7 | 63.4 | 3.868 | 0.006 |
| yes same diversity2 - no same diversity4 | 5.533 | 10.7 | 91 | 0.517 | > 0.5 |
| yes same diversity2 - yes same diversity4 | 0.322 | 10.43 | 110.5 | 0.031 | > 0.5 |
| no diff diversity4 - yes diff diversity4 | 30.839 | 8.6 | 96.5 | 3.587 | 0.0119 |
| no diff diversity4 - no same diversity4 | 2.73 | 10.33 | 179.1 | 0.264 | > 0.5 |
| no diff diversity4 - yes same diversity4 | -2.481 | 10.44 | 131.9 | -0.238 | > 0.5 |
| yes diff diversity4 - no same diversity4 | -28.108 | 10.48 | 128.2 | -2.683 | 0.1373 |
| **yes diff diversity4 - yes same diversity4** | -33.32 | 10.22 | 172.9 | -3.26 | 0.0286 |
| no same diversity4 - yes same diversity4 | -5.211 | 11.95 | 149.9 | -0.436 | > 0.5 |

**Supplementary file 1f**. **Type-I Analysis of Variance Table of the experimental treatment effects on total crop yield per plot (square-root transformed)**

*DenDF*, degrees of freedom of error term; *NumDF*, degrees of freedom of term; *F-value*, variance ratio; *Pr(>F)*, error probability. P-values in bold are signiﬁcant at α = 0.1; . (P < 0.1); * (P < 0.05), ** (P < 0.01), *** (P < 0.001). n=276

|  | *NumDF* | *DenDF* | *F value* | *Pr(>F)* |  |
| --- | --- | --- | --- | --- | --- |
| *Fertilizer* | 1 | 7.682 | 18.5184 | **0.002862** | ****** |
| *History* | 1 | 241.392 | < 1 | > 0.5 |  |
| *Mono vs. mixtures* | 1 | 19.973 | 3.5836 | **0.072934** | **.** |
| *Diversity* | 1 | 19.956 | < 1 | 0.481957 |  |
| *Fertilizer x history* | 1 | 241.338 | < 1 | > 0.5 |  |
| *Fertilizer x mono vs. mix* | 1 | 238.145 | < 1 | > 0.5 |  |
| *Fertilizer x diversity* | 1 | 237.548 | < 1 | > 0.5 |  |
| *History x mono vs. mix* | 1 | 237.451 | < 1 | > 0.5 |  |
| *History x diversity* | 1 | 237.596 | < 1 | > 0.5 |  |
| *Fertilizer x history x mono vs. mix* | 1 | 237.347 | < 1 | > 0.5 |  |
| *Fertilizer x history x diversity* | 1 | 237.487 | < 1 | > 0.5 |  |

**Supplementary file 1g**. **Type-I Analysis of Variance Table of the experimental treatment effects on mean and coefficient of variation of height, per species per plot (species level) in year 3 (2020)**

*DenDF*, degrees of freedom of error term; *NumDF*, degrees of freedom of term; *F-value*, variance ratio; *Pr(>F)*, error probability. P-values in bold are signiﬁcant at α = 0.05; * (P < 0.05), ** (P < 0.01), *** (P < 0.001), n=1726

|  |  | *Mean* | | | *Coefficient of variation* | | |  |
| --- | --- | --- | --- | --- | --- | --- | --- | --- |
|  | *NumDF* | *DenDF* | *F value* | *Pr(>F)* | *DenDF* | *F value* | *Pr(>F)* |  |
| *Fertilizer* | 1 | 7.9 | < 1 | > 0.5 | 7.04 | 16.8065 | **4.51E-03 **** | |
| *History* | 1 | 169.16 | 4.2929 | **0.039789 *** | 197.26 | < 1 | > 0.5 | |
| *Mono vs. mixtures* | 1 | 23.57 | < 1 | > 0.5 | 45.01 | < 1 | > 0.5 | |
| *Diversity* | 1 | 10.68 | < 1 | > 0.5 | 45.48 | < 1 | > 0.5 | |
| *Fertilizer x history* | 1 | 168.93 | < 1 | > 0.5 | 197.18 | 5.8068 | **0.016883 *** | |
| *Fertilizer x mono vs. mix* | 1 | 416.7 | 9.2129 | **0.002554 **** | 487.47 | 5.0102 | **0.025648 *** | |
| *Fertilizer x diversity* | 1 | 118.78 | < 1 | > 0.5 | 127.23 | < 1 | > 0.5 | |
| *History x mono vs. mix* | 1 | 418.29 | 1.435 | 0.231632 | 483.31 | < 1 | > 0.5 | |
| *History x diversity* | 1 | 119.33 | < 1 | 0.418149 | 128.24 | < 1 | > 0.5 | |
| *Fertilizer x history x mono vs. mix* | 1 | 417.97 | < 1 | > 0.5 | 483.3 | < 1 | 0.445694 | |
| *Fertilizer x history x diversity* | 1 | 119.15 | < 1 | > 0.5 | 128.25 | < 1 | > 0.5 | |

**Supplementary file 1h**. **Type-I Analysis of Variance Table of the experimental treatment effects on mean and coefficient of variation of width, per species per plot (species level) in year 3 (2020)**

*DenDF*, degrees of freedom of error term; *NumDF*, degrees of freedom of term; *F-value*, variance ratio; *Pr(>F)*, error probability. P-values in bold are signiﬁcant at α = 0.1; . (P < 0.1); * (P < 0.05), ** (P < 0.01), *** (P < 0.001), n=1726

|  |  | *Mean* | | | *Coefficient of variation* | | |
| --- | --- | --- | --- | --- | --- | --- | --- |
|  | *NumDF* | *DenDF* | *F value* | *Pr(>F)* | *DenDF* | *F value* | *Pr(>F)* |
| *Fertilizer* | 1 | 7.59 | 8.5571 | **0.02027 *** | 7.34 | 4.8793 | **6.12E-02 .** |
| *History* | 1 | 531.5 | 2.0724 | 0.15057 | 233.05 | 2.5024 | 0.11503 |
| *Mono vs. mixtures* | 1 | 19.68 | < 1 | 0.34482 | 534.07 | < 1 | > 0.5 |
| *Diversity* | 1 | 10.47 | < 1 | > 0.5 | 144.7 | < 1 | 0.3493 |
| *Fertilizer x history* | 1 | 530.95 | 1.6511 | 0.19937 | 232.84 | < 1 | > 0.5 |
| *Fertilizer x mono vs. mix* | 1 | 523.5 | 2.5201 | 0.11301 | 533.17 | < 1 | 0.37767 |
| *Fertilizer x diversity* | 1 | 526.73 | 3.905 | **0.04866 *** | 142.23 | < 1 | > 0.5 |
| *History x mono vs. mix* | 1 | 522.6 | < 1 | > 0.5 | 528.21 | 1.4807 | 0.2242 |
| *History x diversity* | 1 | 526.98 | 1.1295 | 0.28837 | 144.44 | 1.1028 | 0.2954 |
| *Fertilizer x history x mono vs. mix* | 1 | 522.34 | < 1 | > 0.5 | 528.23 | 1.7197 | 0.1903 |
| *Fertilizer x history x diversity* | 1 | 526.26 | < 1 | 0.3413 | 144.54 | < 1 | 0.35153 |

**Supplementary file 1j**. **Type-I Analysis of Variance Table of the experimental treatment effects on mean and coefficient of variation of LDMC, per species per plot (species level) in year 3 (2020)**

*DenDF*, degrees of freedom of error term; *NumDF*, degrees of freedom of term; *F-value*, variance ratio; *Pr(>F)*, error probability. P-values in bold are signiﬁcant at α = 0.1; . (P < 0.1); * (P < 0.05), ** (P < 0.01), *** (P < 0.001), n=1726

|  |  | *Mean* | | | *Coefficient of variation* | | |
| --- | --- | --- | --- | --- | --- | --- | --- |
|  | *NumDF* | *DenDF* | *F value* | *Pr(>F)* | *DenDF* | *F value* | *Pr(>F)* |
| *Fertilizer* | 1 | 7.86 | 8.0352 | **0.02239 *** | 186.4 | 12.783 | **4.46E-04 ***** |
| *History* | 1 | 183.84 | 3.5956 | **0.05950 .** | 190.46 | < 1 | > 0.5 |
| *Mono vs. mixtures* | 1 | 19.67 | < 1 | > 0.5 | 516.86 | < 1 | > 0.5 |
| *Diversity* | 1 | 5.24 | < 1 | > 0.5 | 116.92 | < 1 | 0.426449 |
| *Fertilizer x history* | 1 | 181.59 | < 1 | > 0.5 | 190.48 | 3.1314 | **0.078397 .** |
| *Fertilizer x mono vs. mix* | 1 | 467.36 | < 1 | > 0.5 | 515.71 | < 1 | 0.434889 |
| *Fertilizer x diversity* | 1 | 115.57 | < 1 | > 0.5 | 114.6 | 3.8577 | **0.051939 .** |
| *History x mono vs. mix* | 1 | 468.83 | < 1 | > 0.5 | 515.4 | < 1 | > 0.5 |
| *History x diversity* | 1 | 116.59 | 1.0418 | 0.30953 | 114.82 | < 1 | 0.335923 |
| *Fertilizer x history x mono vs. mix* | 1 | 468.38 | < 1 | > 0.5 | 515.36 | 1.6235 | 0.203176 |
| *Fertilizer x history x diversity* | 1 | 116.48 | < 1 | > 0.5 | 114.88 | 1.3649 | 0.24511 |

**Supplementary file 1i.** **Type-I Analysis of Variance Table of the experimental treatment effects on mean and coefficient of variation of SLA, per species per plot (species level) in year 3 (2020)**

*DenDF*, degrees of freedom of error term; *NumDF*, degrees of freedom of term; *F-value*, variance ratio; *Pr(>F)*, error probability. P-values in bold are signiﬁcant at α = 0.1; . (P < 0.1); * (P < 0.05), ** (P < 0.01), *** (P < 0.001), n=1726

|  |  | *Mean* | | | *Coefficient of variation* | | |
| --- | --- | --- | --- | --- | --- | --- | --- |
|  | *NumDF* | *DenDF* | *F value* | *Pr(>F)* | *DenDF* | *F value* | *Pr(>F)* |
| *Fertilizer* | 1 | 7.8 | 9.3229 | **0.01623 *** | 7.7 | 7.4375 | **0.026891 *** |
| *History* | 1 | 203.35 | < 1 | > 0.5 | 224.44 | < 1 | > 0.5 |
| *Mono vs. mixtures* | 1 | 27.81 | 2.1781 | 0.15122 | 39.2 | 1.8674 | 0.179558 |
| *Diversity* | 1 | 10.76 | < 1 | > 0.5 | 12.52 | 3.0841 | 0.103467 |
| *Fertilizer x history* | 1 | 203.8 | < 1 | > 0.5 | 224.52 | < 1 | > 0.5 |
| *Fertilizer x mono vs. mix* | 1 | 428.56 | < 1 | > 0.5 | 415.79 | 7.1378 | **0.007844 **** |
| *Fertilizer x diversity* | 1 | 144.51 | < 1 | > 0.5 | 168.99 | 3.165 | **0.077029 .** |
| *History x mono vs. mix* | 1 | 427.99 | 2.4804 | 0.11601 | 412.72 | < 1 | > 0.5 |
| *History x diversity* | 1 | 146.35 | 1.0093 | 0.31674 | 169.58 | < 1 | > 0.5 |
| *Fertilizer x history x mono vs. mix* | 1 | 427.9 | < 1 | > 0.5 | 412.49 | < 1 | > 0.5 |
| *Fertilizer x history x diversity* | 1 | 146.35 | < 1 | 0.92507 | 169.62 | < 1 | > 0.5 |

**Supplementary file 1k**. **Type-I Analysis of Variance Table of the experimental treatment effects on mean and coefficient of variation of mass per seed, per species per plot (species level) in year 3 (2020)**

*DenDF*, degrees of freedom of error term; *NumDF*, degrees of freedom of term; *F-value*, variance ratio; *Pr(>F)*, error probability. P-values in bold are signiﬁcant at α = 0.1; . (P < 0.1); * (P < 0.05), ** (P < 0.01), *** (P < 0.001), n=1726

|  |  | *Mean* | | | *Coefficient of variation* | | |
| --- | --- | --- | --- | --- | --- | --- | --- |
|  | *NumDF* | *DenDF* | *F value* | *Pr(>F)* | *DenDF* | *F value* | *Pr(>F)* |
| *Fertilizer* | 1 | 210.65 | 4.3651 | **0.03788 *** | 180.24 | < 1 | > 0.5 |
| *History* | 1 | 211.89 | < 1 | > 0.5 | 182.21 | < 1 | > 0.5 |
| *Mono vs. mixtures* | 1 | 20.08 | 10.0297 | **0.00483 **** | 33.75 | 17.0854 | **0.000223 ***** |
| *Diversity* | 1 | 10.77 | 1.3367 | 0.27261 | 8.88 | < 1 | > 0.5 |
| *Fertilizer x history* | 1 | 211.88 | < 1 | 0.4163 | 182.12 | 1.6601 | 0.199224 |
| *Fertilizer x mono vs. mix* | 1 | 493.44 | < 1 | 0.43818 | 474.87 | 1.16 | 0.282008 |
| *Fertilizer x diversity* | 1 | 137.39 | < 1 | > 0.5 | 118.13 | 1.4557 | 0.230024 |
| *History x mono vs. mix* | 1 | 493.59 | 1.4337 | 0.23174 | 470.19 | 4.9519 | **0.026536 *** |
| *History x diversity* | 1 | 138.08 | < 1 | > 0.5 | 119.33 | < 1 | > 0.5 |
| *Fertilizer x history x mono vs. mix* | 1 | 493.59 | 2.7072 | 0.10053 | 470.29 | < 1 | > 0.5 |
| *Fertilizer x history x diversity* | 1 | 138.1 | < 1 | 0.42842 | 119.29 | < 1 | > 0.5 |

**Supplementary file 1l**. **Type-I Analysis of Variance Table of the experimental treatment effects on community-weighted mean and coefficient of variation of height, per plot (community level) in year 3 (2020)**

*DenDF*, degrees of freedom of error term; *NumDF*, degrees of freedom of term; *F-value*, variance ratio; *Pr(>F)*, error probability. P-values in bold are signiﬁcant at α = 0.1; . (P < 0.1); * (P < 0.05), ** (P < 0.01), *** (P < 0.001). n=271

|  |  | *CWM* | | | *Coefficient of variation* | | |  |
| --- | --- | --- | --- | --- | --- | --- | --- | --- |
|  | *NumDF* | *DenDF* | *F value* | *Pr(>F)* | *DenDF* | *F value* | *Pr(>F)* |  |
| *Fertilizer* | 1 | 7.804 | 2.687 | 1.41E-01 | 6.983 | 7.6601 | **2.79E-02 *** | |
| *History* | 1 | 231.321 | < 1 | 0.47728 | 234.457 | 3.9279 | **0.04866 *** | |
| *Mono vs. mixtures* | 1 | 19.976 | < 1 | 0.81612 | 20.095 | 16.9763 | **0.000527 ***** | |
| *Diversity* | 1 | 19.985 | < 1 | > 0.5 | 19.944 | 3.0898 | **0.094124 .** | |
| *Fertilizer x history* | 1 | 228.963 | < 1 | > 0.5 | 234.705 | < 1 | 0.415143 | |
| *Fertilizer x mono vs. mix* | 1 | 227.04 | 5.6668 | **0.01812 *** | 238.629 | 5.7944 | **0.016838 *** | |
| *Fertilizer x diversity* | 1 | 226.105 | < 1 | 0.39255 | 235.462 | < 1 | > 0.5 | |
| *History x mono vs. mix* | 1 | 226.029 | < 1 | > 0.5 | 235.769 | 2.0403 | 0.154507 | |
| *History x diversity* | 1 | 226.301 | < 1 | > 0.5 | 235.616 | < 1 | > 0.5 | |
| *Fertilizer x history x mono vs. mix* | 1 | 226.108 | < 1 | > 0.5 | 236.013 | < 1 | > 0.5 | |
| *Fertilizer x history x diversity* | 1 | 226.151 | < 1 | > 0.5 | 235.639 | < 1 | > 0.5 | |

**Supplementary file 1m**. **Type-I Analysis of Variance Table of the experimental treatment effects on community-weighted mean and coefficient of variation of width, per plot (community level) in year 3 (2020)**

*DenDF*, degrees of freedom of error term; *NumDF*, degrees of freedom of term; *F-value*, variance ratio; *Pr(>F)*, error probability. P-values in bold are signiﬁcant at α = 0.1; . (P < 0.1); * (P < 0.05), ** (P < 0.01), *** (P < 0.001), n =271

|  |  | *CWM* | | | *Coefficient of variation* | | |  |
| --- | --- | --- | --- | --- | --- | --- | --- | --- |
|  | *NumDF* | *DenDF* | *F value* | *Pr(>F)* | *DenDF* | *F value* | *Pr(>F)* |  |
| *Fertilizer* | 1 | 7.484 | 6.0869 | **4.09E-02 *** | 7.352 | 10.7862 | **1.25E-02 *** | |
| *History* | 1 | 233.262 | < 1 | > 0.5 | 239.502 | < 1 | > 0.5 | |
| *Mono vs. mixtures* | 1 | 19.917 | 1.7012 | 0.207003 | 20.153 | 15.6397 | **0.000773 ***** | |
| *Diversity* | 1 | 19.935 | < 1 | > 0.5 | 19.819 | 2.4097 | 0.13641 | |
| *Fertilizer x history* | 1 | 231.77 | 1.6122 | 0.205462 | 239.1 | < 1 | > 0.5 | |
| *Fertilizer x mono vs. mix* | 1 | 228.97 | 11.0067 | **0.001056 **** | 237.11 | 8.7687 | **0.003376 **** | |
| *Fertilizer x diversity* | 1 | 227.323 | 3.9302 | **0.048631 *** | 233.942 | 1.1993 | 0.274588 | |
| *History x mono vs. mix* | 1 | 227.213 | < 1 | > 0.5 | 234.231 | 4.7012 | **0.031149 *** | |
| *History x diversity* | 1 | 227.671 | < 1 | > 0.5 | 234.017 | < 1 | > 0.5 | |
| *Fertilizer x history x mono vs. mix* | 1 | 227.362 | < 1 | > 0.5 | 234.403 | 2.0867 | 0.149924 | |
| *Fertilizer x history x diversity* | 1 | 227.418 | < 1 | > 0.5 | 233.993 | < 1 | 0.427744 | |

**Supplementary file 1n**. **Type-I Analysis of Variance Table of the experimental treatment effects on community-weighted mean and coefficient of variation of SLA, per plot (community level) in year 3 (2020)**

*DenDF*, degrees of freedom of error term; *NumDF*, degrees of freedom of term; *F-value*, variance ratio; *Pr(>F)*, error probability. P-values in bold are signiﬁcant at α = 0.1; . (P < 0.1); * (P < 0.05), ** (P < 0.01), *** (P < 0.001), n=271

|  |  | *CWM* | | | *Coefficient of variation* | | |  |
| --- | --- | --- | --- | --- | --- | --- | --- | --- |
|  | *NumDF* | *DenDF* | *F value* | *Pr(>F)* | *DenDF* | *F value* | *Pr(>F)* |  |
| *Fertilizer* | 1 | 7.612 | < 1 | 3.93E-01 | 7.426 | 3.6336 | **9.59E-02 .** | |
| *History* | 1 | 227.776 | 2.0837 | 0.15025 | 225.431 | 2.4299 | 0.120442 | |
| *Mono vs. mixtures* | 1 | 19.754 | < 1 | > 0.5 | 19.682 | 15.332 | **0.000879 ***** | |
| *Diversity* | 1 | 19.858 | < 1 | > 0.5 | 20.339 | 8.4912 | **0.008482 **** | |
| *Fertilizer x history* | 1 | 227.607 | < 1 | > 0.5 | 224.689 | < 1 | 0.360883 | |
| *Fertilizer x mono vs. mix* | 1 | 225.127 | < 1 | > 0.5 | 222.442 | 7.7108 | **0.005957 **** | |
| *Fertilizer x diversity* | 1 | 223.255 | < 1 | > 0.5 | 222.197 | 3.5889 | **0.059465 .** | |
| *History x mono vs. mix* | 1 | 222.96 | 3.4724 | **0.06371 .** | 220.028 | < 1 | > 0.5 | |
| *History x diversity* | 1 | 223.809 | < 1 | > 0.5 | 222.407 | < 1 | > 0.5 | |
| *Fertilizer x history x mono vs. mix* | 1 | 223.168 | < 1 | > 0.5 | 220.233 | < 1 | 0.465566 | |
| *Fertilizer x history x diversity* | 1 | 223.828 | < 1 | > 0.5 | 221.495 | < 1 | > 0.5 | |

**Supplementary file 1o. Type-I Analysis of Variance Table of the experimental treatment effects on community-weighted mean and coefficient of variation of LDMC, per plot (community level) in year 3 (2020)**

*DenDF*, degrees of freedom of error term; *NumDF*, degrees of freedom of term; *F-value*, variance ratio; *Pr(>F)*, error probability. P-values in bold are signiﬁcant at α = 0.1; . (P < 0.1); * (P < 0.05), ** (P < 0.01), *** (P < 0.001), n=271

|  |  | *CWM* | | | *Coefficient of variation* | | |  |
| --- | --- | --- | --- | --- | --- | --- | --- | --- |
|  | *NumDF* | *DenDF* | *F value* | *Pr(>F)* | *DenDF* | *F value* | *Pr(>F)* |  |
| *Fertilizer* | 1 | 7.809 | 10.5893 | **1.20E-02 *** | 7.368 | 2.1001 | 1.88E-01 | |
| *History* | 1 | 225.249 | 4.3323 | **0.03853 *** | 233.338 | 4.1789 | **0.042053 *** | |
| *Mono vs. mixtures* | 1 | 19.998 | < 1 | > 0.5 | 20.123 | 10.747 | **0.003737 **** | |
| *Diversity* | 1 | 20.012 | < 1 | > 0.5 | 19.46 | < 1 | 0.39891 | |
| *Fertilizer x history* | 1 | 223.935 | < 1 | 0.44016 | 233.38 | < 1 | 0.318908 | |
| *Fertilizer x mono vs. mix* | 1 | 222.756 | < 1 | > 0.5 | 236.325 | 4.6325 | **0.032385 *** | |
| *Fertilizer x diversity* | 1 | 221.841 | < 1 | > 0.5 | 232.776 | < 1 | > 0.5 | |
| *History x mono vs. mix* | 1 | 221.728 | < 1 | > 0.5 | 233.192 | < 1 | > 0.5 | |
| *History x diversity* | 1 | 222.051 | < 1 | > 0.5 | 233 | < 1 | > 0.5 | |
| *Fertilizer x history x mono vs. mix* | 1 | 221.903 | < 1 | > 0.5 | 233.579 | 2.708 | 0.101189 | |
| *Fertilizer x history x diversity* | 1 | 221.981 | < 1 | > 0.5 | 233.206 | < 1 | 0.351866 | |

**Supplementary file 1p**. **Type-I Analysis of Variance Table of the experimental treatment effects on community-weighted mean and coefficient of variation of mass per seed, per plot (community level) in year 3 (2020)**

*DenDF*, degrees of freedom of error term; *NumDF*, degrees of freedom of term; *F-value*, variance ratio; *Pr(>F)*, error probability. P-values in bold are signiﬁcant at α = 0.1; . (P < 0.1); * (P < 0.05), ** (P < 0.01), *** (P < 0.001), n=271

|  |  | *CWM* | | | *Coefficient of variation* | | |  |
| --- | --- | --- | --- | --- | --- | --- | --- | --- |
|  | *NumDF* | *DenDF* | *F value* | *Pr(>F)* | *DenDF* | *F value* | *Pr(>F)* |  |
| *Fertilizer* | 1 | 7.319 | 2.4734 | 0.15792 | 6.612 | 1.0304 | 3.46E-01 | |
| *History* | 1 | 240.762 | < 1 | > 0.5 | 225.443 | 1.4213 | 0.234444 | |
| *Mono vs. mixtures* | 1 | 20.012 | < 1 | > 0.5 | 20.021 | 14.3694 | **0.001145 **** | |
| *Diversity* | 1 | 19.996 | < 1 | > 0.5 | 19.95 | < 1 | 0.474995 | |
| *Fertilizer x history* | 1 | 235.451 | < 1 | > 0.5 | 226.003 | < 1 | > 0.5 | |
| *Fertilizer x mono vs. mix* | 1 | 238.14 | < 1 | 0.33727 | 239.091 | < 1 | > 0.5 | |
| *Fertilizer x diversity* | 1 | 235.395 | < 1 | > 0.5 | 236.191 | < 1 | > 0.5 | |
| *History x mono vs. mix* | 1 | 235.632 | 1.5019 | 0.22161 | 236.507 | 5.4785 | **0.020084 *** | |
| *History x diversity* | 1 | 235.537 | < 1 | > 0.5 | 236.403 | < 1 | > 0.5 | |
| *Fertilizer x history x mono vs. mix* | 1 | 235.858 | 3.5455 | **0.06094 .** | 236.836 | < 1 | > 0.5 | |
| *Fertilizer x history x diversity* | 1 | 235.548 | < 1 | > 0.5 | 236.483 | < 1 | > 0.5 | |

**Supplementary file 1q**. **Type-I Analysis of Variance Table of the experimental treatment effects on functional richness in year 3 (2020)**

*DenDF*, degrees of freedom of error term; *NumDF*, degrees of freedom of term; *F-value*, variance ratio; *Pr(>F)*, error probability. P-values in bold are signiﬁcant at α = 0.1; . (P < 0.1); * (P < 0.05), ** (P < 0.01), *** (P < 0.001). n=271

|  | *NumDF* | *DenDF* | *F value* | *Pr(>F)* |
| --- | --- | --- | --- | --- |
| *Fertilizer* | 1 | 240.322 | 12.1182 | **0.000593 ***** |
| *History* | 1 | 239.331 | < 1 | 0.490791 |
| *Diversity* | 2 | 20.095 | 224.8931 | **1.76E-14 ***** |
| *Fertilizer x history* | 1 | 239.229 | < 1 | > 0.5 |
| *Fertilizer x diversity* | 2 | 240.165 | 1.6086 | 0.202324 |
| *History x diversity* | 2 | 239.277 | < 1 | > 0.5 |
| *Fertilizer x history x diversity* | 2 | 239.303 | < 1 | > 0.5 |

**Supplementary file 1r**. **Type-I Analysis of Variance Table of the experimental treatment effects on FPAR in year 3 (2020)**

*DenDF*, degrees of freedom of error term; *NumDF*, degrees of freedom of term; *F-value*, variance ratio; *Pr(>F)*, error probability. P-values in bold are signiﬁcant at α = 0.05; * (P < 0.05), ** (P < 0.01), *** (P < 0.001). n=2484

|  | *NumDF* | *DenDF* | *F value* | *Pr(>F)* |
| --- | --- | --- | --- | --- |
| *Fertilizer* | 1 | 7.76 | 18.986 | **0.002604 **** |
| *History* | 1 | 2420.53 | 15.3962 | **8.96E-05 ***** |
| *Mono vs. mixtures* | 1 | 20.03 | 1.3841 | 0.253196 |
| *Diversity* | 1 | 19.98 | < 1 | > 0.5 |
| *Fertilizer x history* | 1 | 2421.07 | 1.0275 | 0.310837 |
| *Fertilizer x mono vs. mix* | 1 | 2445.01 | 2.2105 | 0.137204 |
| *Fertilizer x diversity* | 1 | 2443.87 | < 1 | > 0.5 |
| *History x mono vs. mix* | 1 | 2443.67 | < 1 | 0.483969 |
| *History x diversity* | 1 | 2444.07 | < 1 | > 0.5 |
| *Fertilizer x history x mono vs. mix* | 1 | 2442.98 | < 1 | > 0.5 |
| *Fertilizer x history x diversity* | 1 | 2443.39 | < 1 | > 0.5 |

**Supplementary file 1s**. List of species mixture combinations.

| *Monoculture* | *2-species mixtures* | *2-species mixtures* | *4-species mixtures* |
| --- | --- | --- | --- |
| Avena | Avena-Lens | Lens-Linum | Avena-Lens-Linum-Coriandrum |
| Triticum | Avena-Linum | Lens-Camelina | Avena-Lens-Camelina-Coriandrum |
| Lens | Avena-Camelina | Lens-Coriandrum | Triticum-Lens-Linum-Coriandrum |
| Linum | Avena-Coriandrum | Linum-Coriandrum | Triticum-Lens-Camelina-Coriandrum |
| Camelina | Triticum-Lens | Camelina-Coriandrum |  |
| Coriandrum | Triticum-Linum | Triticum-Coriandrum |  |
|  | Triticum-Camelina |  |  |
